# Electroendocytosis Is Driven by the Binding of Electrochemically Produced Protons to the Cell’s Surface

**DOI:** 10.1371/journal.pone.0050299

**Published:** 2012-11-27

**Authors:** Nadav Ben-Dov, Inna Rozman Grinberg, Rafi Korenstein

**Affiliations:** Department of Physiology and Pharmacology, Faculty of Medicine, Tel-Aviv University, Tel-Aviv, Israel; BioScience Project, United States of America

## Abstract

Electroendocytosis involves the exposure of cells to pulsed low electric field and is emerging as a complementary method to electroporation for the incorporation of macromolecules into cells. The present study explores the underlying mechanism of electroendocytosis and its dependence on electrochemical byproducts formed at the electrode interface. Cell suspensions were exposed to pulsed low electric field in a partitioned device where cells are spatially restricted relative to the electrodes. The cellular uptake of dextran-FITC was analyzed by flow cytometery and visualized by confocal microscopy. We first show that uptake occurs only in cells adjacent to the anode. The enhanced uptake near the anode is found to depend on electric current density rather than on electric field strength, in the range of 5 to 65 V/cm. Electrochemically produced oxidative species that impose intracellular oxidative stress, do not play any role in the stimulated uptake. An inverse dependence is found between electrically induced uptake and the solution’s buffer capacity. Electroendocytosis can be mimicked by chemically acidifying the extracellular solution which promotes the enhanced uptake of dextran polymers and the uptake of plasmid DNA. Electrochemical production of protons at the anode interface is responsible for inducing uptake of macromolecules into cells exposed to a pulsed low electric field. Expanding the understanding of the mechanism involved in electric fields induced drug-delivery into cells, is expected to contribute to clinical therapy applications in the future.

## Introduction

Electropermeabilization of the cell membrane by pulsed high electric fields has been used in the last three decades to induce the uptake of molecules, in particular DNA, into the intracellular compartment of the cell [1]. The excepted paradigm in this research field has been that electropermeabilization occurs via short-lived pores in the plasma membrane that are formed when the cross-membrane potential difference reaches a threshold value of ∼200 mV [2], [3], [4]. However, the mechanism for uptake of large molecules, DNA in particular, has not been fully resolved [5]. Since the 1990s, numerous reports have shown that in addition to electropermeabilization, the application of high electric fields can induce endocytic-like processes [6], [7], [8], [9], [10], [11]. The fact that both plasma membrane permeabilization and membrane vesiculation can occur during and following cell exposure to the high electric fields, has hindered the identification of the mechanism(s) underlying electroendocytosis. A significant advance in exploring this phenomenon was the discovery that exposure of cells to non-permeabilizing pulsed train of low electric fields (LEF), leads to a stimulated uptake of different fluid phase and adsorptive fluorescent probes of low and high molecular weight via endocytic-like pathway [12], [13]. The exposure to LEF was reported to generate an alteration of cell surface, leading to elevated adsorption of macromolecules such as bovine serum albumin (BSA), dextran and DNA, as well as to an enhanced uptake [14]. This surface alteration, attributed to the electrophoretic segregation of charged mobile lipid and protein entities in the cell plasma membrane, was suggested to be responsible both for enhanced adsorption and stimulated uptake, via change of the plasma membrane curvature that enhances budding processes [14]. Recently, an important development in the understanding of the mechanism that underlies endocytic-like uptake was reported, revealing that high concentration of hydrogen ions at the cells’ surface can induce inward membrane vesiculation and uptake of macromolecules [15].

Since the exposure of cells to high or low electric fields, which leads to electropermabilization and electroendocytosis, involves direct contact between the electrodes and the cells’ medium, the cells are expected to be exposed to electrochemical byproducts created at the electrode-solution interface. In the present study we examined the involvement of radical oxygen species (ROS) and elevation in hydrogen-ion concentrations in the uptake of macromolecules induced by low pulsed train of electric fields. Our finding reveals that uptake only occurs at the proximity of the anode and that electrochemical acidification of the extracellular media, is sufficient to enhance the uptake of macromolecules, including DNA, by cells via endocytic-like pathway.

## Materials and Methods

### Chemicals

Karnovsky fixative (x2 stock): 6% paraformaldehyde, 1% glutaraldehyde in 0.2M cacodylate buffer. Sodium ascorbic acid (SAA), Bis-Dehydroascorbic acid (DHA), BSA-FITC, dextran-FITC (38 kD with 0.005 FITC/glucose ratio and 71 kD with 0.01 FITC/glucose ratio), dextranase, propidium iodide (PI), hydrochloric acid (HCl), lucifer yellow (LY) tetramethyl-benzidine (TMB), 4,6-Diamidino-2-phenylindole dihydrochloride (DAPI), nigericin and hank’s balanced salt solution (HBSS) were purchased from Sigma-Aldrich (Rechovot, Israel). Trypan-blue (TB), phosphate buffered saline (PBS) and 4-(2-Hydroxyethyl) piperazine-1-ethanesulfonic acid (HEPES) were purchased from Biological industries (Beit Haemek, Israel). Dichlorodihydrofluorescein diacetate (H_2_DCF-DA) and Cholera toxin subunit B conjugated with Alexa 488 were purchased from Invitrogen (Ca. USA).

### Cell Culture

COS-7 cells (ATCC No. CRL-1651, monkey kidney fibroblast-like cells) and HaCaT cells (human keratinocyte [16]) were cultured in Dulbecco’s Modified Eagle Medium (DMEM), supplemented with L-glutamine (2 mM), 10% FCS and 0.05% PSN solution. All cells were grown in 75 cm^2^ tissue culture flasks (Corning) at 37°C, in a humid atmosphere of 5% CO_2_ in air. Cells were harvested before reaching ∼80% confluence by using 0.25% trypsin solution (with 0.05% EDTA) for 5 min at 37°C. The cells were centrifuged (1 min at 400 g), their solution aspirated and then re-suspended in growth medium. All culture media, antibiotics, trypsin and serum products were purchased from Biological Industries (Beit Haemek, Israel).

### Exposure Set-up for Low Electric Fields

**Figure 1:**
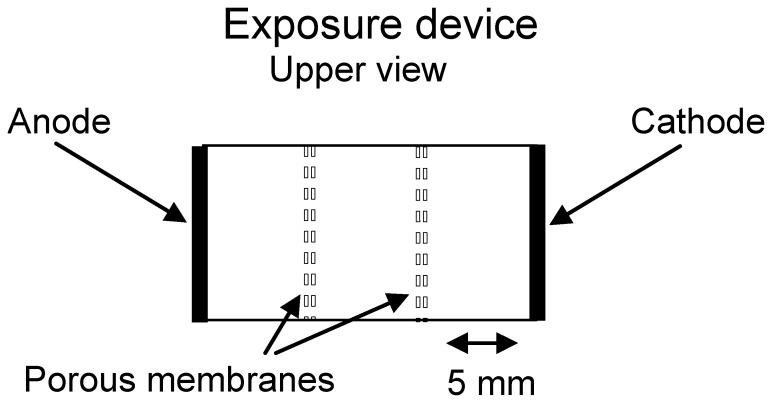
Schematic representation of the three partitions exposure device. The device consists of two Pt electrodes positioned on the extreme sides of a rectangular chamber. The chamber is partitioned by two porous membranes of low electrical resistance, forming three distinct and equal compartments of the anode, cathode and a central one in between.

Exposure of cells to low-intensity trains of unipolar rectangular voltage pulses was carried out by employing an electric pulse generator (Grass S44 Stimulator, West Warwick, USA). Exposure of cells in a three-compartment exposure set-up [17] consisted of a rectangular chamber made from polystyrene, 15 mm long, 10 mm wide, with 0.5 cm^2^ area planar platinum electrodes positioned on the extreme sides (Fig. 1). The chamber is partitioned by two porous membranes (polyethersulfone, 0.8 µm pores and 200 µm thickness) into three distinct and equal compartments: anode, cathode and a central one. The passage of charged low MW fluorescent probes from the anode or cathode compartments into the central one was monitored using tryphan-blue (TB) or Lucifer-yellow (LY) by measuring their fluorescence (480/590 nm or 430/530 nm, respectively). The passage of hydrogen ions and oxidative intermediates into the central compartment was monitored by a pH electrode or by TMB color conversion, respectively.

The electric field parameters applied were monitored on-line by recording the voltage and the current (by means of a wide band current probe, Pearson) using an oscilloscope. Typically, a cell suspension is exposed to a train of low electric fields (LEF) consisting of unipolar rectangular pulses with duration of 180 µs, frequency of 500 Hz for the total time of exposure of one minute. The temperature of the solution during exposure was measured using fiberoptic temperature sensors (FISO Technologies, Quebec, Canada). The measured mean transient temperature rise at the end of a 1 min exposure of cells to LEF of 20V/cm at 24°C was 1.4±0.6°C (mean ± SD).

### Uptake Studies

Cells were harvested, re-suspended in DMEM (glucose 1 mg/ml, FCS 5%), and incubated (37°C, 5% CO_2_) as designated by the experimental setup. When no pre-treatments were required, the cells were kept in the DMEM for the same duration. Following incubation period, the cells were centrifuged at 400 g for 1 min, the solution aspirated, and cells were re-suspended in HBSS.

Cell suspensions (1×10^6^ cells/ml, 0.5 ml per compartment) were exposed to LEF in the three-compartment device in the presence of dextran-FITC for 60 s at room temperature (∼24°C). The samples were then immediately diluted with 5 fold larger volume of ice-cold DMEM-H (25 mM HEPES supplemented DMEM without phenol red) and kept on ice. Exposure of cells to pulses of low pH was done through titration of HBSS with HCl for 60 s, terminated by the addition of 3x volume of cold DMEM-H. Cells in the control group were incubated with dextran-FITC for 60 s at room temperature, after which they were moved to ice as described above. Each experiment was repeated independently at least 9 times spread over three different days.

### Removal of the Dextran-FITC fraction Adsorbed to Cell Surface

**Figure 2:**
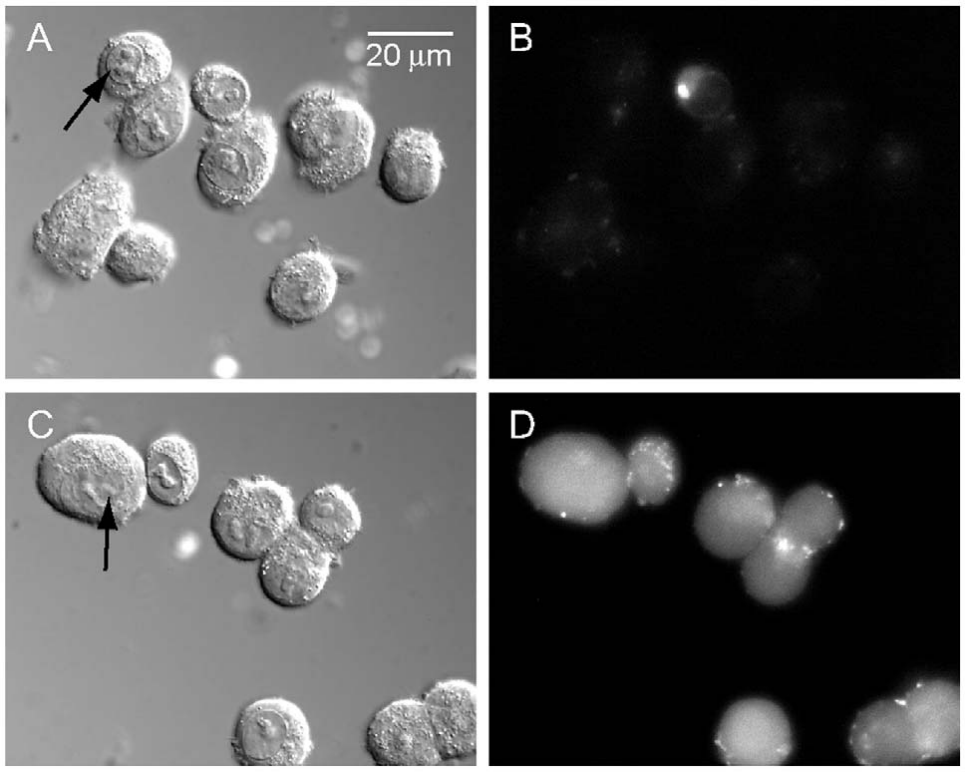
Cell images following LEF induced uptake of dextran-FITC. COS-7 cells were exposed to LEF in the anode compartment for 60 s in the presence of dextran-FITC (38 kD). The cells were washed as described in section 2.5 and mounted on glass slide in PBS containing 0.01% TB. Images of cells were acquired by SCLM in the DIC (images A and C. arrows point to the cell nucleus) and fluorescence (485/530 nm, images B and D) modes. Images A and B depict cells exposed to LEF at ∼4°C. Images C and D depict cells exposed to LEF at room temperature.

For determining the fraction of the fluorescent dextran adsorbed to the cells’ membrane, cell suspensions were pre-cooled to 4°C and exposed to LEF for 60 s in the presence of dextran, thereby eliminating the contribution of endocytosis. For measuring the cell auto-fluorescence, LEF exposure was carried out in the absence of the external fluorescent probe. In preparing for analysis, the cells were washed twice in DMEM-H medium by centrifugation (400 g for 1 min, Sorvall RT6000D, Du-Pont USA). To further remove remaining dextran adsorbed to the cell membrane, the cell suspensions were centrifuged again, supernatant aspirated and the pellet re-suspended with 0.5 ml DMEM-H supplemented with 2 unit/ml of dextranase for 10 min at 24°C. Cells were washed again and the cell pellet was re-suspended with 0.2 ml DMEM-H. Shortly before FACS analysis, PI (25 µg/ml) as a label for membrane-permeable cells and TB (0.01% w/v) as a fluorescence quenching agent for extracellular FITC [18] were added to each sample. Microscopic examination of the cells verifies the absence of detectable fluorescent corona around the cell membrane (Fig. 2).

### Flow Cytometry (FACS)

Flow cytometry analysis was carried out by FACSort (Becton @ Dickson, San Jose, CA), employing a 488 nm argon laser excitation. The green fluorescence of FITC was measured via 530/30 nm band filter, the red fluorescence of PI was measured via a 585/42 nm band filter. To eliminate signals due to cellular fragments, only those events with forward scatter and side scatter comparable to whole cells were analyzed. Ten thousand cells were examined for each sample and data were collected in the list mode. The analysis of flow cytometry data was performed using WINMDI 2.9 software (Joe Trotter, The Scripps Research Institute). For each sample the geometrical mean was calculated without including PI stained cells. The efficiency of probe loading was characterized by cell labeling intensity using fold of induction, (FI) that was calculated as the ratio of the geometric mean of fluorescence in experimental samples to that of control non-exposed ones.

### Determination of Oxidative Stress in Solution

Extracellular media analysis was carried by employing TMB, a colorless solution that is transformed into blue when oxidized. TMB was added to the solution before or after its exposure to LEF and the solution collected and stabilized with 1.25 M HCl, which transforms its color to yellow. Optical density of the stabilized oxidation product was measured at 450 nm employing a microplate reader (GENius, Tecan, USA).

### Determination of Intracellular Oxidative Stress

Preloading cells with 10 µM of Carboxy-H_2_DCFDA was performed by incubating the cells (at 37°C in humid atmosphere with 5% CO_2_) for 90 min in serum-free DMEM. H_2_DCF-DA is essentially a cell-permeable, non-fluorescent molecule that can be transformed by the action of intracellular esterases to a charged, cell non-permeable H_2_DCF molecule. In this form the molecule can be oxidized, mainly by super oxides and radical hydroxyls to the green fluorescent dichlorofluorescein (DCF) [19]. Harvested cells were incubated and loaded with carboxy-H_2_DCF-DA. Shortly after the loading process the cells were washed once with HBSS before being exposed to the electric field under the designated experimental setup. Following the exposure, cell suspensions were washed and prepared for FACS analysis as described. All washing steps were performed with ice cold solutions.

### Quantitative and Qualitative Evaluation of Solution’s pH

pH analysis in the medium shortly after LEF exposure was determined by pH electrode (SevenEasy, Mettler). For qualitative evaluation of the transient pH formed at the anode surface during the application of electric current, we used irreversible pH sensitive paper indicators (PANPEHA, Sigma-aldrich) prepared by cutting 5 mm by 7 mm rectangular section from the original paper strips. The pH indicator paper was placed perpendicularly to the electrode surface plane and in one quick movement dipped in and pulled out from the anodic compartment solution followed by gentle placement on a blotting paper.

### Osmolarity

Solutions were analyzed employing an osmometer (Vapro 5520, Wescor), based on the evaporation point method.

### Medium Conductivity

Conductivity was lowered by replacing some of the soluble salt ions with sucrose. 300 mM sucrose solution in water was used for diluting HBSS at several ratios to final sucrose concentrations from 200 mM down to 50 mM.

### Plasmid DNA

A 4.7 kb pAcGFP1-C1 plasmid, (Clontech, Takara), which encodes a GFP reporter gene, was used for the DNA transfection and uptake studies. The DNA was prepared using PureYield™ Plasmid Midiprep System (Promega), according to manufacturer’s protocol and dissolved in nuclease free water. The quantity and quality of the plasmid was assessed using NanoDrop ND-1000 spectrophotometer (Thermo Scientific) by light absorption at 260/280 nm ratio and by 1% agarose gel electrophoresis. For uptake studies the plasmids were labeled with Cy3 using the Label IT® Nucleic Acid Labeling Kit, Cy3 (Mirus), according to the manufacturer’s protocol. Positive control transfections were performed using TurboFect™ (Fermentas) transfection reagent according to the manufacturer’s protocol.

### Microscopy

Scanning Confocal Laser Microscope (SCLM; LSM 410, Zeiss, Germany) was used for acquiring microscopic cell images. In SCLM, computer-generated images of 0.5 µm optical sections were obtained at the approximate geometric center of the cell as determined by repeated optical sections. The images were processed by axiovision software (Zeiss, Germany) and image annotation was made using Illustrator CS software (Abode, USA).

### Statistical Analysis

Statistical analyses were performed by student’s t-test and one way ANOVA, using Microsoft Excel.

## Results

### Electrically Induced Uptake is not Uniform in between the Electrodes

In order to differentiate between the contributions of the electric field itself and its electrochemical byproducts to the extent of uptake, we constructed an exposure system where the cells could be maintained in three compartments; anodal, central and cathodal (Fig. 1). These compartments are separated by two highly porous (0.8 µm) and tortuous membranes. These membranes possess very low electric resistance when placed in physiological solutions (device resistance is 47.6 Ω without the membranes and 50 Ω with both of them), yet are able to delay the diffusion time between the compartments due to internal tortuous structure which provides a longer travel path for the passing molecules. This was experimentally verified using two low molecular weight dyes (LY and TB) which showed no traceable penetration across the membrane barriers in the three compartment exposure system, following one minute of exposure to LEF. COS-7 cell suspensions were placed in the three compartment exposure device employing Pt electrodes. Uptake was induced by exposure to LEF (20 V/cm, 200 mA/cm^2^, 180 µs pulse width, 500 Hz repetition rate) for 60 s at 24°C, in the presence of dextran-FITC (38 kD, 0.1 mM). Following their exposure, the cells were washed by PBS and analyzed by FACS. Cells exposed to LEF in the anodal compartment showed an ∼3 fold increase in cellular uptake of dextran-FITC relative to the constitutive uptake in the absence of LEF (Table 1, P<0.05 by t-test, n = 9). However, no enhanced uptake was found in cells present in the other two compartments, i.e. the central and cathodal ones.

**Table 1:** Comparison of cellular uptake and electrochemical byproducts in the three compartments of the exposure device.

Compartment	Uptake^a^	ROS^b^ (DCFintensity)	pH^c^
**Anode**	3.14±1.53	4.94±2.22	5.5
**Center**	1.07±0.08	0.71±0.32	7.5
**Cathode**	1.09±0.12	0.76±0.33	12

a Uptake of Dextran-FITC in terms of exposed/constitutive uptake.

b ROS determined by DCF relative fluorescence in terms of exposed/unexposed cells.

c Measured in HBSS after its exposure to LEF.

### Electrically Induced Uptake Depends on Current Density more then on Electric Field Strength

**Figure 3:**
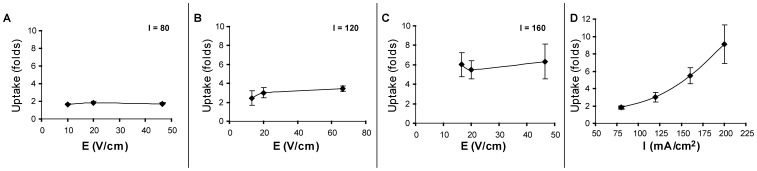
The extent of dextran-FITC uptake as a function of electric field strength and current density. Uptake of 71 kD dextran-FITC by COS-7 cells was performed in the anodal compartment and analyzed by FACS. Results are given as folds of fluorescence geometrical mean ± SD of LEF induced uptake relative to the constitutive uptake. (A to C) Uptake experiments were performed at different constant current densities of I = 80 mA/cm^2^ (A); I = 120 mA/cm^2^ (B); I = 160 mA/cm^2^ (C), as function of electric field strength (P>0.05 by one-way ANOVA for each current density with n = 12). (D) Uptake experiments were performed at a constant electric field (20 V/cm) as a function of electric current densities (P<0.05 by one-way ANOVA, n = 12).

The relative contribution of the electric field and the electric current to the elevated uptake of dextran-FITC was further examined by exposing COS-7 cells to LEF in dextran solutions possessing different electrical conductivities. In order to improve assay sensitivity, we employed a 71 kD dextran (0.1 mM) possessing a higher number of FITC conjugates per molecule. Conductivity was altered by substituting NaCl with sucrose while maintaining iso-osmotic conditions. Data is presented only for the cells exposed in the anode compartment, since cells in the other compartments (i.e. cathodal and central) showed no enhanced uptake over the constitutive level. The results reveal that variation in electric field strength has minor impact on cellular uptake of dextran-FITC at a given current density (Fig. 3A, B and C). Explicitly, Fig. 3D describes the uptake as function of electric current density (at 20 V/cm) possessing a second order polynomial dependency.

### Electrically Induced Uptake is Independent of Electrochemically Produced ROS

Electric current is known to promote oxidation at the anode interface through the production of reactive oxidative intermediates. The term oxidative stress (OS) refers to an imbalance between cellular production of reactive oxidative species (ROS) and their disintegration, leading to elevation of intracellular oxidative activity and consequent damage. Such OS, either extracellular or intracellular, could play a role in the electrically induced uptake. In order to study this possibility we first examined the formation of ROS in the three-compartment exposure set-up. ROS was monitored by measuring the colorimetric oxidation of TMB present in solution during its exposure to LEF. TMB in the anodic compartment solution changed its color during electric current application, but addition of TMB immediately after LEF termination failed to produce any color conversion. Supplementing the solution with 2 mM sodium ascorbic acid (SAA), prior to its exposure to LEF in the anodic compartment was sufficient to prevent TMB oxidation during the exposure (Table 2). The intracellular OS was monitored by the fluorescence intensity of DCF of cells preloaded with H_2_DCF. Only cells exposed to LEF in the anode compartment developed enhanced fluorescence intensity (Table 1). COS-7 cells exposed to LEF in the presence of an antioxidant (2 mM SAA), possessed 66% less DCF fluorescence intensity (Table 2). Cells were loaded with the intracellular antioxidant DHA by incubation in glucose free DMEM with 5% FCS and 1 mM DHA for 90 min. Once in the cytosol, DHA, the stable reduced form of ascorbic acid, is converted to ascorbic acid by intracellular enzymes [20], [21]. Pre-loading the cells with DHA before their exposure to LEF was sufficient to completely abolish the DCF fluorescence increase (Table 2).

**Table 2:** The effect of anti-oxidants on the cellular oxidative stress and dextran uptake following exposure to LEF in the anodic compartment.

Additives	TMB^a^	DCF^b^	Uptake^c^
Non	2.29±0.06	5.13±1.98	9.13±2.22
[SAA] (1 mM)	0.30±0.03	3.60±1.57	-
[SAA] (2 mM)	0.05±0.02	1.57±1.05	7.72±1.45
[DHA] (1 mM)	N.A.	0.29±0.55	7.60±2.49

a O.D. (450 nm) of solution.

b Relative cell fluorescence in terms of exposed/unexposed cells.

c Uptake of Dextran-FITC in terms of exposed/constitutive uptake.

In order to determine the role played by OS in LEF induced uptake, COS-7 cells exposure to LEF was carried out in the presence of dextran-FITC (71 kD, 0.1 mM). Dextran uptake was compared between cells in standard HBSS, cells in HBSS supplemented with 2 mM SAA and cells preloaded with DHA. These minor modifications of HBSS have no impact on the solution’s original pH (pH 7.4) and osmolarity (290 mOs). The presence of antioxidants at concentrations that we found to abolish OS had no statistically significant effect on the extent of dextran-FITC uptake by the cells (Table 2, P>0.05, by one-way ANOVA, n = 9).

### Dependence of Electrically Induced Uptake on Acidification in the Anode Compartment

Electrolysis of water is known to be responsible for acidification near the anode and alkalization near the cathode. However, the exposure of HBSS supplemented with 100 mM HEPES to LEF in the anode compartment, produced no steady-state changes in the solution’s pH (pH 7.4) or osmolarity (290 mOs), relative to its original values, when measured after LEF termination. The exposure of HBSS without additional buffering shows that the solution’s pH is shifted with dependence on the electrode, i.e. alkaline near the cathode and acidic near the anode (Table 1). In order to monitor the transient pH change occurring at the anode’s surface during the exposure to LEF, an irreversible pH-sensitive paper indicator was dipped-in perpendicularly to the electrode surface and pulled out immediately. This method verified the existence of a transient drop in spatial pH profile near the anode interface down to the range of 1.0–2.0 units.

**Figure 4:**
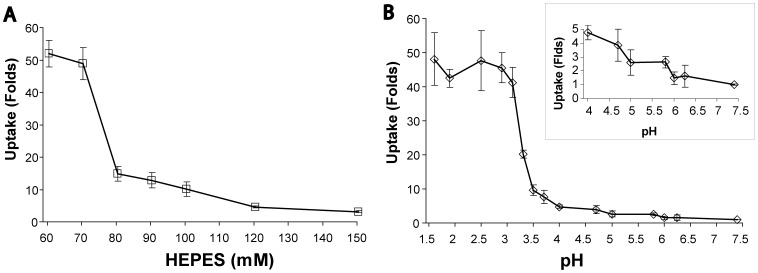
Dextran-FITC uptake as function of solution’s buffer capacity or solution’s pH. Uptake of 71 kD dextran-FITC by COS-7 cells was analyzed by FACS. Results are given as folds of fluorescence geometrical mean ± SD of induced uptake relative to constitutive uptake. (A) Cells were exposed to LEF in the anode compartment for 60 s at different concentrations of HEPES buffered solutions. There is a statistically significant difference between all adjacent points (P<0.05 by *t*-test), except between the 60 mM and the 70 mM (P>0.05 by *t* test). (B) Cells exposed for 60 s to solutions tittered to different pH, in the presence of 71 kD dextran-FITC. The insert depicts a better detailed view in the pH range between 7.4 and 4.0. A statistically significant difference from the constitutive cell uptake begins at pH 5.8 (2.6 fold, P<0.01 by *t*-test, n = 9) down to pH 1.6. Cells’ uptake did not change significantly between pH 3.0 and pH 1.6 (P>0.05, one way ANOVA, n = 9).

In order to validate the role of the transient acidification during exposure in the enhanced uptake by LEF we varied the concentration of HEPES buffer (60 mM to 150 mM) in the exposure solution (HBSS), in the presence of dextran-FITC (71 kD, 0.1 mM). Solution’s iso-osmolarity was maintained by replacing NaCl with HEPES and current density of 200 mA/cm^2^ was kept constant for all experimental instances. Fig. 4A reveals that reducing the buffer concentration in the exposure solution strongly increased the extent of dextran-FITC uptake by COS-7 cells in a sigmoid-like plot, having the steepest slope occurring between 80 mM and 70 mM HEPES.

For simulating the effect of electrochemical acidification on the cellular uptake of dextran-FITC, COS-7 cells were suspended in 10 mM MES buffered HBSS, and the solution’s pH was lowered by titration with hydrochloric acid. Following 60 s exposure to external low pH in the presence of dextran-FITC (71 kD, 0.1 mM), the cell suspensions were immediately diluted with excess volume of cold DMEM to restore the normal pH 7.4. Fig. 4B reveals the dependence of cellular uptake on external pH level. This dependence is characterized by a gradual linear increase from pH 7.0 to pH 4.0, with a steep sigmoid-like elevation in uptake in the range of pH 4 to pH 3.

### Low pH Induces the Entry of Naked DNA Plasmid into Cells

**Figure 5:**
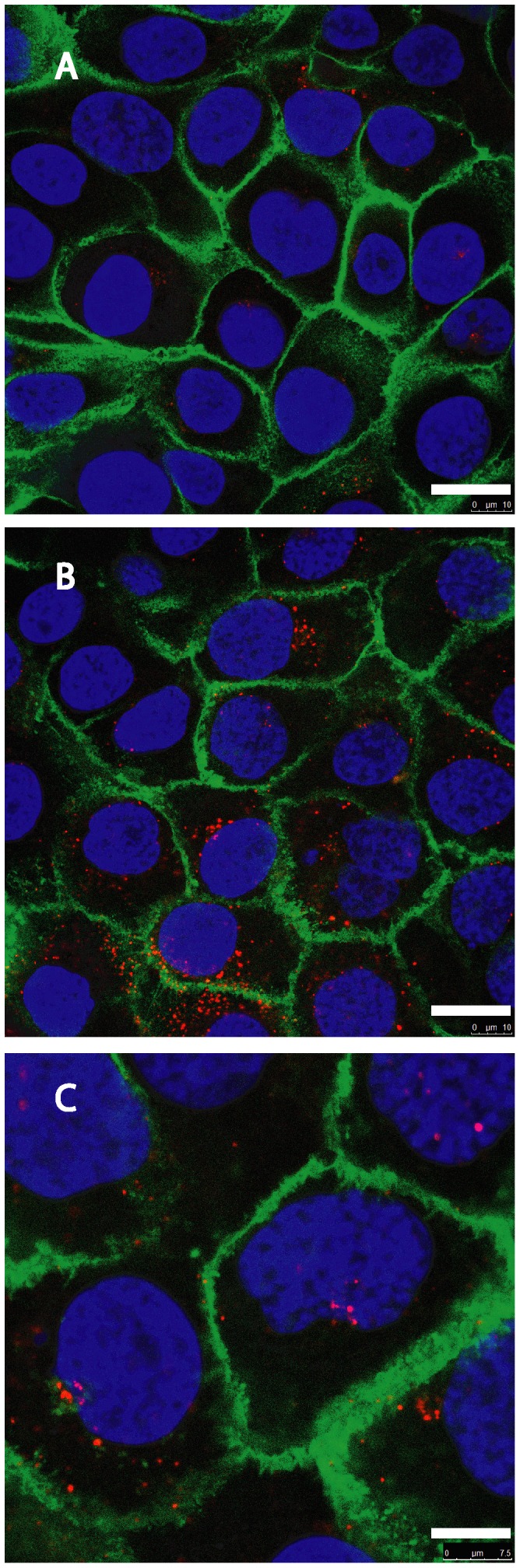
Representative cell images of plasmid DNA uptake, induced in a solution of low pH. HaCaT cells were incubated in the presence of Cy3 tagged DNA plasmid (red), at either pH 7.4 or pH 5.0. Cell membrane is stained with alexa-488 (green) conjugated subunit B cholera toxin, and the nucleus is stained with DAPI (blue). (A) Cells incubated at pH 7.4, show very little presence of tagged plasmid. Bar size = 20 µm. (B) Cells incubated at pH 5.0, showing extensive presence of the plasmid in the cells. Bar size = 20 µm. (C) Higher magnification of cells incubated at pH 5.0. Bar size = 10 µm.

Cultures of HaCaT cells were incubated in either HBSS (pH 7.4) or HBSS with 20 mM MES (tittered to pH 5) in the presence of 0.6 nM (0.1 µg/well) plasmid tagged by Cy3 for a period of 45 min at 24°C. Following treatment, the cells were prepared for SCLM analysis by fixation with karnovsky’s solution, followed by 3 washes with PBS and further staining of the cell’s surface and nucleus with Cholera toxin subunit-B (0.2 µg/ml, Alexa 488 conjugated) and 1 µg/ml DAPI, respectively. Confocal fluorescence imaging was used to demonstrate the uptake of the Cy3 tagged DNA plasmid at pH 5, compared to uptake at pH 7.4 (Fig. 5).

For the study of DNA transfection efficiency, cultures of HaCaT cells were incubated for 1 hr in either HBSS (pH 7.4) or HBSS with 20 mM MES buffer (tittered to pH 5) in the presence of 4.8 nM (3 µg/well) plasmid. The duration of the incubation was limited by the tendency of the cells to detach from the culture surface in response to prolonged exposure to low pH. Following their incubation with the DNA plasmid, the cultures were washed with HBSS and incubated in a growth medium for 48 hours. The extent of transfection by the plasmid was measured by employing FACS for counting the fraction of cells that express the GFP fluorescent protein. The potency of the plasmid was verified by inducing its transfection in the cells by standard TurboFect™ (Fermentas) protocol. Despite the apparent entry of plasmids into the cells under low pH conditions, only a very small fraction (∼0.1%) of the cells expressed the encoded fluorescent protein, similar to the transfection level found in the control cells, exposed to the plasmid at pH 7.4.

## Discussion

Electroendocytosis, an electric-field induced endocytic-like process, was previously reported to enable the uptake of macromolecules by cells [12], [13], [14]. In the present study we examine the possibility that electroendocytosis is driven by the formation of electrolytic byproducts.

The impact of the cells’ location relative to the electrodes on the extent of uptake was examined in an exposure device where the cells are restricted to one of three partitions – near the anode, near the cathode or at 5–10 mm from the electrodes. When employing this exposure set-up, essentially the same electrical field prevails in all three compartments. However, no increase in cellular uptake was observed in the central and the cathodal compartments, whereas the cells in the anodal one demonstrate an impressive increase of uptake (See Table 1). This finding does not comply with an effect driven by an electric field. Furthermore, it is shown that the enhanced uptake depends on the electric current density rather than on the electric field strength (Fig. 3). All together, these findings imply that the electrically induced uptake is more likely to be associated with the electrochemical byproducts formed in the anodic compartment than with the electric field *per-se*. This conclusion points out a clear mechanistic difference from that of electroporation, which depends on electric field strength. A differentiation between LEF induced uptake and an electroporation driven one was already previously discussed [12]. The main difference between electroporation and inward vesiculation is the fact that the later phenomenon does not involve membrane permeability change. One way to demonstrate the existence of permeability change of the cell membrane for small molecules is to load the cells with the small molecules and to examine whether they undergo enhanced efflux following exposure to electric field. Such studies have been carried out previously [12] and no enhanced efflux was detected.

Electrolysis at the anode-solution interface produces radical oxidative species as well as increased concentration of hydrogen ions. We show that electrochemical oxidative intermediates which are formed in the anodal compartment are instrumental in elevating the intracellular oxidative stress. However, while the presence of extracellular and intracellular antioxidants can attenuate this cellular oxidative stress, it conveys no significant impact on the extent of electrically induced uptake.

Electrohydrolysis is the decomposition of water due to an electric potential applied across a pair of electrodes. If electrohydrolysis occurs in low water conductivity, H^+^ cations and OH^−^ anions will remain at the interface of their respective electrodes. This will lead to electrode polarization and unless a very large electric potential is applied to cause an increase in water ionization, the electrolysis will be slowed down. If the conductivity of the water is raised through a dissociated electrolyte (e.g. NaCl), the electrolyte anions and cations neutralize the buildup of charges at the electrodes interface, allowing for the flow of electricity and the continued hydrolysis. Thus while the electrode potential drives the water decomposition, its rate depends on the current that pass through the electrodes [22].

Anodic hydrolysis generates a marked decrease in the local pH at the anode interface. Due to the high buffer capacity of the medium, this local elevation of hydrogen ion concentration has a transient nature, diminishing both temporally and spatially [23]. Thus, cells located away from the anode interface are expected to be less susceptible to the effect of electrochemically produced acidity. The dependence of LEF induced uptake on buffer capacity, presented in Fig. 4A, leads us to propose that an elevated concentration of hydrogen ions is the major contributor to the phenomenon of electrically induced uptake.

This dependence of uptake on extracellular proton concentration is in-line with our finding that electroendocytosis shows higher response to anodal current density than to electric field. Protons are produced by anodal electrolysis of water. In our experimental setup, higher current densities are associated with higher solution conductivities and these electrical parameters are responsible for the translocation of hydrogen ions away from the anode interface into the bulk solution and their rate of production at the anode. Therefore higher current densities would be expected to raise both protons production and their accessibility to the cells.

We simulated the electrochemical low pH environment by chemically exposing the cell suspension to a low pH in the range of 7.4 to 1.7 (Fig. 4B). The extent by which the cells internalize dextran-FITC as a function of buffer capacity or pH possess a sigmoid-like curve. These findings are in line with our recent report [15] where we show (by electron microscopy) that the exposure of the cell’s surface to extracellular low pH promotes the formation of endocytic-like membrane invaginations into the cytoplasm and detail the kinetics of the enhanced uptake of macromolecules into the cells. This conclusion is also supported by a previous study [12], showing that the endocytic routes, consisting of clathrin-dependent pathways and caveolin-dependent pathway, contribute very little to the LEF-induced uptake.

Low pH has been shown before to induce inward membrane tubules in lipid vesicles [24] and to induce the formation of plasma membrane vesicle and uptake of macromolecules in cells [15]. The mechanism that enforces a membrane to invaginate was suggested to rely on elevated concentration of extracellular hydrogen ions which bind to the anionic charged sites on the external membrane leaflet. This would result in local reduction of charge density on the external membrane surface, with consequent increase in local cross-membrane charge asymmetry. Such a state would yield a negative value for the spontaneous curvature of the plasma membrane and impose upon it an inward bending [25], [26]. An inward bending of the plasma membrane is the initiating event of endocytic-like processes [27] which consequently lead to the formation of a membrane connected vesicle and finally the scission of the bud neck and separation of the vesicle from the mother membrane.

Two studies of electroendocytosis have suggested a dependence of cellular uptake on the strength of the applied electric field [7], [28], seemingly contradicting our results. As we pointed earlier, the electric potential is responsible for the decomposition of water and the production of hydrogen ions at the anode. Therefore, under experimental conditions of constant current density in a conductive medium (such as biological solutions), electric field would be correlated with enhanced hydrolysis. However, as the distribution of ions into the solution is less efficient at low current densities, the effect of protons on cells’ membrane (and consequent uptake) is restrained (note the trendline in Figs. 3B and 3C). In the study reported by Mahrour et al. [28], a bipolar signal was chosen to avoid electrophoresis and to minimize electrochemical reactions at the electrodes. However the extent of this “minimized” contribution and the transient pH changes in the proximity of the electrodes were not reported. Furthermore, the role of the medium’s conductivity and buffer capacity were not studied with relation to the cell’s uptake. In fact, it is reported that under mild alkaline pH, the stimulatory effect of the electric field on uptake was lost [28]. In a recent study by Lin et al. [7], electroendocytosis was shown to constitute endocytis-like processes, but its dependence on the medium’s conductivity, buffer capacity or transient pH changes was not examined.

The possible involvement of endocytic-like uptake in electroporation has been reported before. In one important example, DNA was efficiently taken up by large unilamellar vesicles exposed to a short pulse of electric field as a result of the electro-stimulated formation of endosome-like vesicles rather than via field-induced membrane pores [29]. Other studies have elucidated the involvement of endocytosis in the uptake of plasmid DNA, as a complementary process to the absorption of DNA to the plasma membrane by the applied electric field. Translocation of these DNA complexes through the membrane is implied to occur after, rather than during, electric pulse application [6], [7], [8], [9], [10], [11]. It should be pointed that under our experimental protocol, the cells’ suspension was cooled immediately after the termination of electric pulses, leaving very little time for endocytosis to commence.

As electroporation is associated with lowered pH at the anodic front [23], the consequent cells’ membrane protonation may play part in the insertion of DNA into cells. This assumption was examined in the present study by exposing the cells to pH 5 in the presence of a naked plasmid DNA. The results, shown in Fig. 5, demonstrate that the plasmids have entered the cells and could indeed be found in their cytoplasm following exposure to low pH. However, very few cells were able to successfully undergo transfection by this method. This outcome is in line with previous reports showing that the mere entry of naked DNA into the cytoplasm is not sufficient for the induction of transfection, mainly due to the nuclear barriers that need to be crossed [30].

### Conclusions

The present study reveals that uptake induced by pulsed low electric field (electroendocytosis) is restricted to the vicinity of the anode. The extent of the uptake depends on anodic current density more than on electric fields possessing values <65 V/cm. Our experimental findings show that the major component to induce such uptake is the production of surplus hydrogen ions by anodic hydrolysis. This type of uptake is possibly an inherent companion of electroporotion, the practice of which involves acidification of the solution near the anode.
